# To Keep or Not to Keep? The Hamletic Umbilical Dilemma: Preservation versus Reconstruction of the Umbilicus in Vertical Abdominoplasty

**DOI:** 10.3390/jcm12010078

**Published:** 2022-12-22

**Authors:** Giuseppe Nisi, Martino Giudice, Stefano Bacchini, Giorgio Fasano, Luigi Verre, Roberto Cuomo, Luca Grimaldi

**Affiliations:** 1 Department of Medicine, Surgery and Neuroscience—Plastic and Reconstructive Surgery Unit, University of Siena, 53100 Siena, Italy; 2 Department of Medicine, Surgery and Neurosciences—Surgical Oncology Unit, University of Siena, 53100 Siena, Italy

**Keywords:** umbilicus, abdominoplasty, plastic surgery, post-bariatric surgery, umbilical reconstruction

## Abstract

(1) Background: The role of the umbilical scar and its repositioning remains one of the most important surgical steps in the execution of any type of abdominoplasty, including those involving “inverted-t” or “fleur de lys” incisions. A consequence of this is a surgeon’s Hamletic dilemma: to keep or not to keep the original umbilical scar? (2) Methods: A retrospective observational study was conducted on all patients undergoing “T-inverted” abdominoplasty at the Department of Plastic Surgery of the Santa Maria alle Scotte University Hospital, Siena, between January 2018 and December 2020. Twelve months after the surgery we submitted to all patients the U-score questionnaire about their feelings about their umbilicus’s appearance. Patients could assign a score from 1 (very dissatisfied) to 4 (very satisfied) to each of the five items of the score. (3) Results: The average of the scores attributed by the nine patients in whom the navel was preserved is 13 (Range 10–17), while in patients on whom a navel reconstruction was performed, the mean score is 16.8 (Range 12–20). The mean score of patients with a reconstructed umbilicus is, therefore, statistically higher than that of the other group of patients (*t*-value = 3.88, *p* = 0.000374) with an average increase of 3.8 points. (4) Conclusions: We can state that the reconstruction of a new navel is the right answer to the Hamletic dilemma in patients having undergone vertical or anchor abdominoplasty.

## 1. Introduction

The abdominal region plays a fundamental role in defining the harmony of the body profile. The fact of being a vessel for new life places the abdomen in a condition of visibility and unique centrality. Over the centuries, its artistic representation has also undergone considerable variations, starting from the Venus of Willendorf, passing through the diaphanous medieval virgins in which it is rigorously hidden from view by long robes and slightly protruding as a sign of fertility, to the re-explosion of fat, redundant and roughly exposed forms of the Renaissance, up to its modern flat, athletic, sculpted and almost androgynous version, but which still maintains its attractiveness, mystery and sensuality intact. A key role in the region’s aesthetics is represented by the umbilical scar, which defines its center, space and harmony. Itis the scar “par excellence”, the primal cut, the sign that perpetuates the mystery of creation and life, the ancestral connection with the womb and through it with the world, the eternal memory of everyone’s genesis.

It is so important that it may be present even where it should not be, even in those who, unknown to the mother’s womb, had their creation directly through God, the Gods or part of them. It is, therefore, a small detail, a miserable scar that explains an innate necessity in the human mind: to humanize what is divine and, at the same time, divinize what is human. Thus, here is Buonarroti’s Adam while with his finger outstretched, he brushes the divine forefinger to receive, from the Creator, that life that his unnatural navel in the foreground says he had in another way; or Venus, who, if as the legend tells she was born from the foam generated by contact with the water of the blood and the seed of Uranus, not only has a navel, but one painted to perfection by Botticelli with careful brushstrokes and positioned to serve as the centerpiece of the scene. After all, it is always the navel, also, at the center of science, which acts as a hinge to the circle within which to inscribe the perfection of man, postulated by Vitruvius and brought to life by the genius hands born in Vinci.

Perhaps this can explain why abdominoplasty is one of the most requested and most performed Aesthetic Plastic Surgery operations in general, and, in particular, after the significant and sudden weight losses induced by bariatric surgery. It is precisely in the morpho-functional subversion of the abdominal region, the effect of sudden weight loss, that the plastic surgeon finds his natural battlefield. The challenge, in fact, is great, and is represented by having to carry out a real reconstruction of the region in its entirety, complicated by the natural heterogenicity and variety of clinical presentations, and by the need to perform large detachments and voluminous dermo-adipose removals, all through long and often multiple skin incisions. The surgeon’s task is, therefore, to recreate an entire part of the body, to restore the most natural harmony of volumes and dimensions, to alternate concavity and convexity, leaving the slightest visible trace of his work, or better, not leaving any of them. In the post-bariatric patient, all this becomes more difficult by the need to add, at times, to the easily concealable horizontal suprapubic skin incision, also a vertical median xyphopubic one.

The role of the umbilical scar and its repositioning remains one of the most important surgical procedures during the execution of any type of abdominoplasty, including those involving “inverted-t” or “fleur de lys” incisions. In fact, in this type of abdominoplasty, the navel falls exactly on the vertical incisional line and is, by the latter, incorporated. In the post-bariatric patient, further characteristics of the navel and of the periumbilical region, such as a greater length, the frequent presence of omphalitis, the finding of potential hernial pathology, and the presence of scars from previous surgical laparoscopic or laparotomic interventions can generate further difficulties at the time of its repositioning. Added to this is the need to combine a straight incision with a curvilinear one, which, although not presenting technical difficulties, very often results in a not-particularly-favorable aesthetic result that negatively affects the overall perception of the abdomen.

Thus, the surgeon’s dilemma becomes Hamletic: to keep the original umbilical scar, accepting the possibility of an unsuitable aesthetic result, or sacrifice it and proceed with its reconstruction, with the awareness that, in the event of failure, an important aesthetic subunit will be lost?

To find an answer to this question, we therefore examined all the vertical abdominoplasty operations performed at our Centre from 2018 to 2020, to determine whether the best aesthetic result was obtained by maintaining the primordial navel or by reconstructing it.

## 2. Materials and Methods

A retrospective observational study was conducted on all patients undergoing “fleur de lys” abdominoplasty at the Department of Plastic Surgery of the Santa Maria alle Scotte University Hospital, Siena, between January 2018 and December 2020. The inclusion and exclusion criteria are summarized in Table 1.

During the planning of the surgery, many factors were considered when choosing between navel reconstruction and navel preservation. In the decision-making process, we first considered the aesthetic preferences of the patients. We also considered the presence of scars in the paraumbilical region, the presence of hernias or pathological conditions affecting the navel, and the quality of the tissue in the paraumbilical region. If pathological circumstances were present, we emphasized that umbilical reconstruction was safer. The native umbilicus, in contrast, was retained in patients with scarring near the base of the two neo-umbilical flaps.

**Figure 1:**
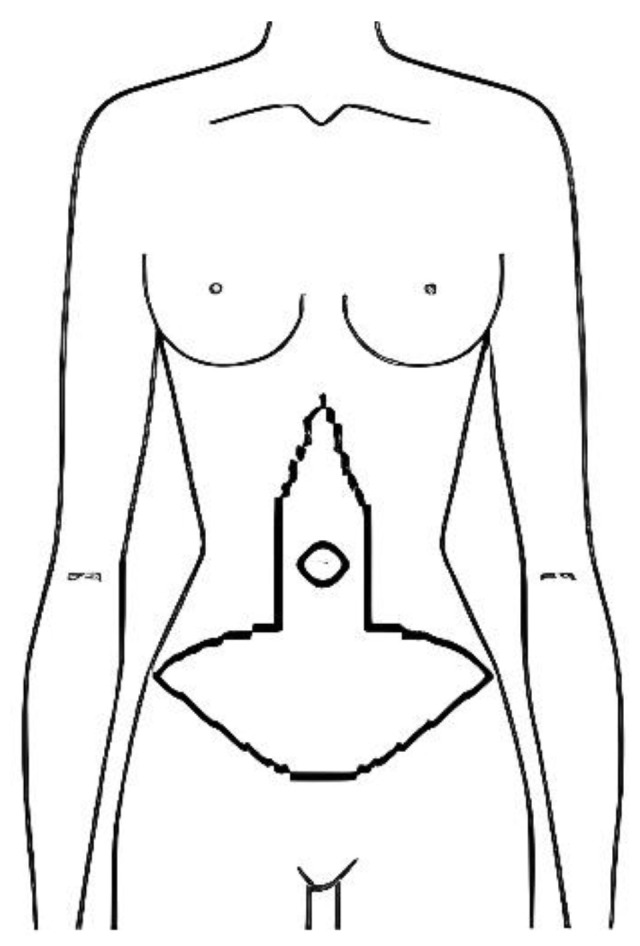
Preoperative drawing of fleur de lys abdominoplasty.

After discussing all these issues with the patient, we agreed on the best choice of intervention and all patients underwent vertical abdominoplasty. In the fleur de lys technique, the classical low horizontal incision is combined with excision of a vertical ellipse of abdominal tissue (Figure 1). This procedure is frequently used in patients in whom there has been a large loss of weight as it allows elimination of both horizontal and vertical skin and tissue excess.

In patients in whom the umbilicus was preserved, the greatest care was taken to preserve the umbilical stalk, so that the umbilicus could be repositioned in its entirety. In contrast, in patients whose navel was reconstructed, the umbilical stalk was amputated and fixed to the muscle fascia.

In patients selected for umbilical reconstruction we decided to use the “two rectangular lateral skin flaps technique”, a technique proposed by Franco in 2006, because of its durable cosmetic results and simplicity to perform.

Twelve months after the surgery we submitted to all patients a questionnaire, proposed by our working team and named U-Score, about their feelings about their umbilicus’s appearance (Table 2). The patient could assign a score from 1 (very dissatisfied) to 4 (very satisfied) to each of the 5 items of the score. 

### Surgical Technique for the Umbilical Reconstruction

Once the length and the width of the vertical flap to be removed were identified, two counterposed rectangular flaps, whose limbs were 2.5 cm, were marked at an average distance of 13 cm (+/− 2 cm depending on the physical characteristics of patients) from the point where the horizontal and the vertical incisions met, in order to reconstruct the neo-navel in the most similar position to the native one.

**Figure 2:**
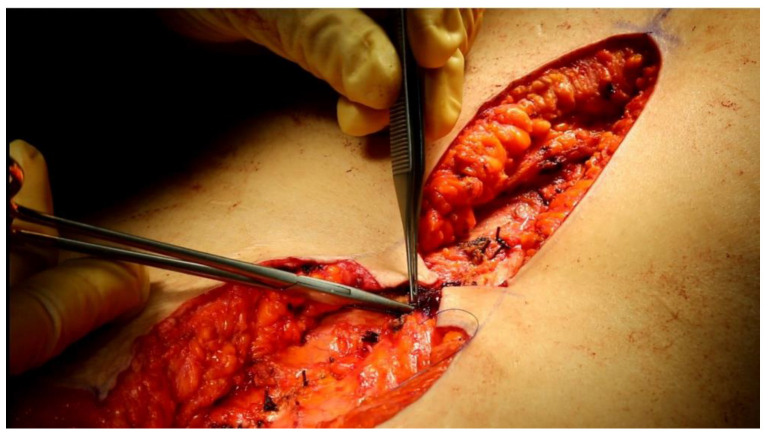
Two rectangular flaps are sutured on the muscular fascial layer.

**Figure 3:**
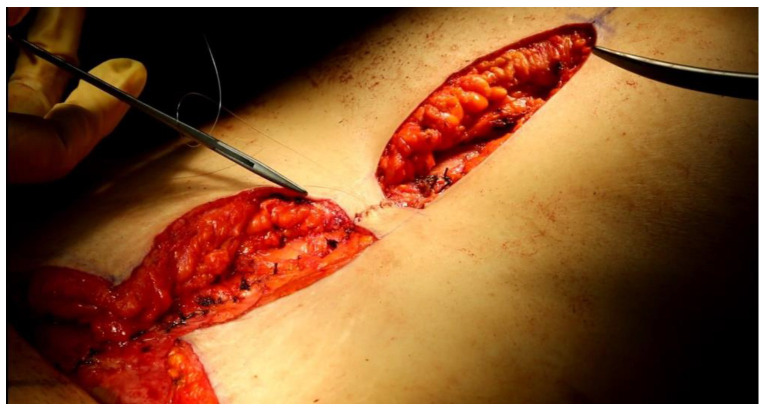
The two rectangular flaps are sutured to each other along the midline.

The two flaps were thinned by removing most of the adipose subcutaneous tissue. At this point the flaps were sutured on the muscular fascial layer (Figure 2) and each other along the midline with 3–4 monofilament non-absorbable 4/0 stitches. The cutaneous limbs at the midline were sutured with stitches in monofilament absorbable 5/0 (Figure 3).

**Figure 4:**
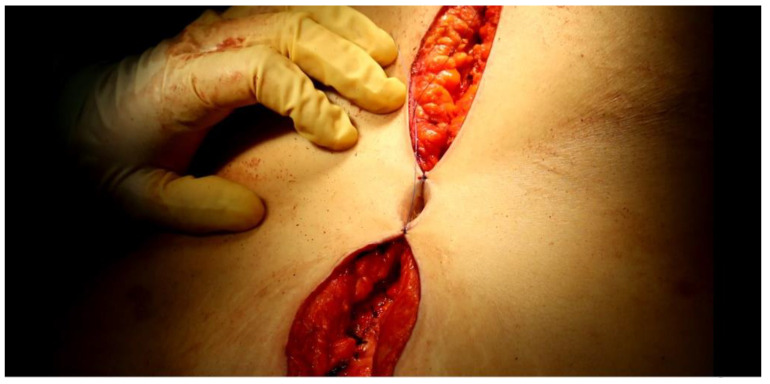
The new navel is now complete.

We then proceeded to the progressive suture of the subcutaneous tissue on the minor sides and finally of the upper and lower corners of the two flaps in absorbable polyfilament 3/0, completed using skin suture in non-absorbable monofilament 3/0. Thus, introflexion and plication of the two flaps were obtained and with them the packaging of the new navel (Figure 4), in which a small swab was housed to be removed after about a week.

## 3. Results

From January 2018 to December 2020 twenty-four patients underwent abdominoplasty with “fleur de lys” incision. In nine of these patients the native navel was preserved and repositioned, while in the other sixteen, the navel was reconstructed according to the “two rectangular lateral skin flaps technique”. Patients’ characteristics are summarized in Table 3. 

The mean age was 35.6 ± 12.4 years (range 20–70 years) and the mean BMI was 26.5 ± 2.35 kg/m² (range 23.1–30 kg/m²). All patients enrolled in the study were ex-obese patients who had undergone bariatric surgery: fourteen of these underwent sleeve gastrectomy, seven underwent gastric bypass and three underwent OAGB. The mean weight loss before the abdominoplasty was 49.7 ± 14.3 kg (range 26–90 kg).

**Figure 5:**
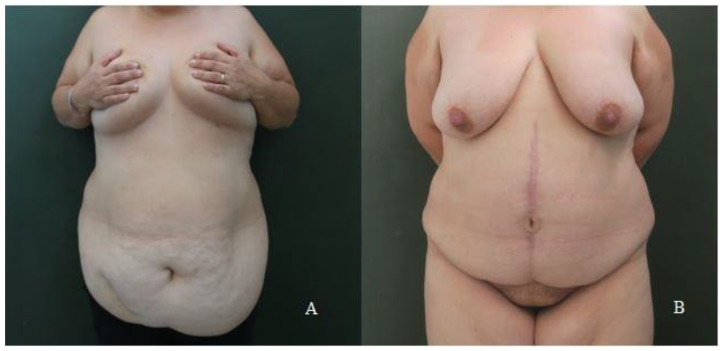
(**A**) A preoperative photo of a patient undergoing vertical abdominoplasty with preservation of the native umbilicus. (**B**) The same patient 12 months after surgery.

**Figure 6:**
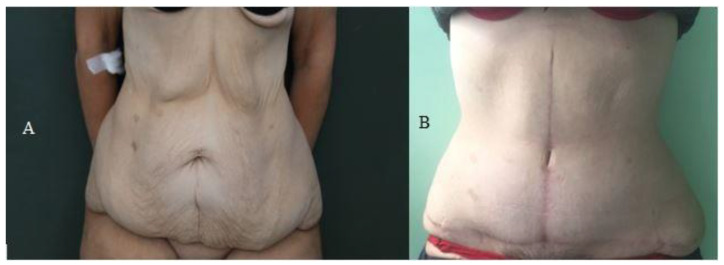
(**A**) A preoperative photo of a patient undergoing vertical abdominoplasty with reconstruction of a new umbilicus. (**B**) The same patient 12 months after surgery.

The satisfaction of each patient was assessed by administering the U-Score questionnaire 12 months after the intervention. Evaluating the sum of the single items of the U-score, reported in Table 4, it can be noted that the average of the scores of the nine patients in whom the navel was preserved (Figure 5) was 13 (Range 10–17), while in patients on whom a navel reconstruction was performed (Figure 6), the mean score was 16.8 (Range 12–20). The mean score of patients with a reconstructed umbilicus was, therefore, statistically higher than that of the other group of patients (*t*-value = 3.88 *p* = 0,000374) with an average increase of 3.8 points.

## 4. Discussion

The umbilicus is an important characteristic of the abdominal wall and neoumbilicoplasty is an essential phase of abdominoplasty. Vary authors have proposed different neoumbilicoplasty techniques in order to obtain an aesthetically pleasing navel. So far, there is not a general agreement on the ideal navel shape and position in abdominoplasty; however, the vertical oval shape, the superior hooding and the absence of protrusion are considered to be more attractive [1,2,3]. In addition, different authors identify a pleasing position at about 2/3 of the distance from the pubis to the xiphisternum [3,4].

During vertical or anchor abdominoplasty, the umbilicus can be treated with two options: umbilicoplasty techniques, with the preservation and transposition of the native navel in the vertical scar [5,6,7,8,9]; or neoumbilicoplasties, with the surgical reconstruction of the navel [10,11,12,13]. In relation to these possibilities, the Hamletic dilemma rises.

A careful assessment of the navel’s preoperative clinical condition and a discussion with the patient before the surgery are mandatory to obtain the most correct answer to the dilemma. Also, the post-bariatric patient’s navel shows additional characteristics that create difficulties for its transposition: a higher average length than a normal-weight patient’s navel, the frequent incidence of omphalitis and hernias and the presence of scars due to previous surgical treatments.

Generally, both surgeons and patients tend to prefer neoumbilicoplasty to the traditional umbilicoplasty [14]. In fact, most navels obtained with neoumbilicoplasty techniques have a vertical oval shape and absence of protrusion. Otherwise, navels transposed with traditional umbilicoplasty techniques appear rounded, wider and frequently protruded [15].

Regarding the reconstruction of the navel, over the years different techniques of neo-umbilicoplasty have been proposed. Lee et al. [16] proposed the Four flaps technique, in which an X-shaped line is incised, creating a depression with the three non-superior flaps, whereas the remaining superior flap is used to form the superior hooding. This technique is easy to perform, yields satisfactory superior hooding and results in a minimal external scar. Shinohara et al. [17] described the Inverted C-V flap, with the C part corresponding to the lower half of the ring and the two V parts extending laterally. For this technique, the most important advantage is the ease of execution. In addition, the scars are oriented horizontally, corresponding to the relaxed tension lines. Watanabe et al. [18] illustrated the Rabbit head-shaped scar skin flap. The main problem of this technique is that the circular scar surrounding the neo-umbilicus tends to contract, making the navel smaller. The Spiral rotational flap presented by Featherstone and Cuckow [19] is based on the tabularization of a skin flap. It gives an excellent long-lasting aesthetic result. The Dome procedure, as illustrated by Senturk et al. [20], is performed to create three skin flaps forming a dome shape to reconstruct the umbilicus.

In our experience, we performed the two lateral pedicle flaps technique on patients having undergone neoumbilicoplasty. This technique was described by Franco in 2006 [10] and by different authors later [14,21]. It is our personal preference. In fact, it shows various advantages, such as being simple to perform and yielding satisfactory and durable cosmetic results. In addition, in the case of vertical abdominoplasty, it enables a new umbilicus without surrounding scars, reducing the possibility of postoperative retraction and reduction. It also reduces the tension along the vertical scar, showing a better aesthetic result and diminishing the risk of a hypertrophic scar.

## 5. Conclusions

Comparing the reconstruction of a new navel and the preservation of the native one, we prefer the first option as an answer to the Hamletic dilemma in patients having undergone vertical or anchor abdominoplasty. In fact, on the U-Score questionnaire, the average score of patients in whom the navel was reconstructed was higher than that obtained in patients in whom the navel was preserved.

## Figures and Tables

**Table 1:** Inclusion and Exclusion Criteria.

Inclusion Criteria	Exclusion Criteria
Obese patients who have undergone at least one bariatric surgery (OAGB, Gastric bypass, Sleeve gastrectomy) with stable weight for at least 12 months Patients with BMI ≤ 30 kg/m^2^ Pittsburgh Rating Scale Score ≥ 10	Patients with BMI ≥ 30.1 kg/m^2^ Pittsburgh Rating Scale Score ≤ 9 Patients with diabetes Patients who are active smokers Patients with unstable weight in the last 12 months Patients who have previously undergone abdominoplasty

**Table 2:** The U-Score.

U-Score Reconstructed Umbilicus	U-Score Native Umbilicus
How satisfied do you feel with new umbilicus?	How satisfied do you feel with native umbilicus?
How do you feel about the shape of the new umbilicus?	How do you feel about the shape of the native umbilicus?
How do you feel about the vertical scar in relation to the new umbilicus?	How do you feel about the vertical scar in relation to the native umbilicus?
How do you feel about the position of the new umbilicus?	How do you feel about the position of the native umbilicus?
How do you feel with the new umbilicus in social instances?	How do you feel with the native umbilicus in social instances?

**Table 3:** Patients’ Characteristics.

	Age	BMI	Weight Loss (Kg)	Type of Bariatric Surgery	Umbilicus
**Patient 1**	48	26.8	48	Sleeve gastrectomy	Native
**Patient 2**	47	23.8	43	One anastomosis gastric bypass	Native
**Patient 3**	31	25.1	58	Sleeve gastrectomy	Native
**Patient 4**	36	23.6	44	Gastric bypass	Native
**Patient 5**	47	24.7	59	Gastric bypass	Native
**Patient 6**	51	26.9	51	Sleeve gastrectomy	Native
**Patient 7**	52	27.1	53	Sleeve gastrectomy	Native
**Patient 8**	25	24.8	68	Sleeve gastrectomy	Native
**Patient 9**	29	25.3	41	Sleeve gastrectomy	Native
**Patient 10**	50	30.0	29	Sleeve gastrectomy	Reconstructed
**Patient 11**	23	29.3	52	One anastomosis gastric bypass	Reconstructed
**Patient 12**	29	23.9	26	Sleeve gastrectomy	Reconstructed
**Patient 13**	28	30.0	39	Gastric bypass	Reconstructed
**Patient 14**	29	28.1	46	Sleeve gastrectomy	Reconstructed
**Patient 15**	28	25.4	67	Sleeve gastrectomy	Reconstructed
**Patient 16**	70	29.2	48	Sleeve gastrectomy	Reconstructed
**Patient 17**	22	27.9	33	Gastric bypass	Reconstructed
**Patient 18**	23	23.4	45	One anastomosis gastric bypass	Reconstructed
**Patient 19**	39	23.5	57	Sleeve gastrectomy	Reconstructed
**Patient 20**	36	29.1	67	Sleeve gastrectomy	Reconstructed
**Patient 21**	22	30.0	47	Gastric bypass	Reconstructed
**Patient 22**	30	23.1	55	Gastric bypass	Reconstructed
**Patient 23**	41	28.8	90	Sleeve gastrectomy	Reconstructed
**Patient 24**	20	27.2	27	Gastric bypass	Reconstructed
**Average**	35.6	26.5	49.7		

**Table 4:** Evaluation with the U-score questionnaire.

	U-Score Reconstructed Umbilicus	U-Score Native Umbilicus	
**Patient 1**		13	
**Patient 2**		11	
**Patient 3**		10	
**Patient 4**		17	
**Patient 5**		14	
**Patient 6**		14	
**Patient 7**		12	
**Patient 8**		10	
**Patient 9**		16	
**Patient 10**	18		
**Patient 11**	18		
**Patient 12**	12		
**Patient 13**	19		
**Patient 14**	16		
**Patient 15**	16		
**Patient 16**	20		
**Patient 17**	15		
**Patient 18**	17		
**Patient 19**	18		
**Patient 20**	20		
**Patient 21**	15		
**Patient 22**	17		
**Patient 23**	14		
**Patient 24**	17		
**Average**	16.8	13	+ 3.8 (*p* = 0.000374)

## Data Availability

The data presented in this study are available on request from the corresponding author. The data are not publicly available due to privacy reasons.
